# Purinergic Receptor Blockade with Suramin Increases Survival of Postnatal Neural Progenitor Cells In Vitro

**DOI:** 10.3390/ijms22020713

**Published:** 2021-01-12

**Authors:** Alejandro Herrera, Sara Morcuende, Rocío Talaverón, Beatriz Benítez-Temiño, Angel M. Pastor, Esperanza R. Matarredona

**Affiliations:** 1 Departamento de Fisiología, Facultad de Biología, Universidad de Sevilla, 41012 Seville, Spain; alejandrohd92@hotmail.com (A.H.); smorcuende@us.es (S.M.); bbtmino@us.es (B.B.-T.); ampastor@us.es (A.M.P.); 2 Instituto de Neurociencias de Castilla y Léon (INCYL), Universidad de Salamanca, 37007 Salamanca, Spain; rtalaveron@usal.es

**Keywords:** purinergic signaling, apoptosis, neural progenitor cells, cell therapy, suramin

## Abstract

Neural progenitor cells (NPCs) are self-renewing and multipotent cells that persist in the postnatal and adult brain in the subventricular zone and the hippocampus. NPCs can be expanded in vitro to be used in cell therapy. However, expansion is limited, since the survival and proliferation of adult NPCs decrease with serial passages. Many signaling pathways control NPC survival and renewal. Among these, purinergic receptor activation exerts differential effects on the biology of adult NPCs depending on the cellular context. In this study, we sought to analyze the effect of a general blockade of purinergic receptors with suramin on the proliferation and survival of NPCs isolated from the subventricular zone of postnatal rats, which are cultured as neurospheres. Treatment of neurospheres with suramin induced a significant increase in neurosphere diameter and in NPC number attributed to a decrease in apoptosis. Proliferation and multipotency were not affected. Suramin also induced an increase in the gap junction protein connexin43 and in vascular endothelial growth factor, which might be involved in the anti-apoptotic effect. Our results offer a valuable tool for increasing NPC survival before implantation in the lesioned brain and open the possibility of using this drug as adjunctive therapy to NPC transplantation.

## 1. Introduction

Neural progenitor cells (NPCs) are self-renewing cells with the ability to differentiate into the three major lineages of the central nervous system: neurons, astrocytes, and oligodendrocytes [1]. During nervous system development, neurons and macroglial cells are formed from NPCs located in the neuroepithelium of the neural tube. After birth, NPCs remain in most mammals only in two neurogenic regions: the subventricular zone (SVZ), adjacent to the lateral ventricles, and the subgranular zone of the dentate gyrus in the hippocampus [1,2,3]. NPCs from postnatal and adult brains can be expanded in vitro in the presence of epidermal growth factor (EGF) and fibroblast growth factor 2 (FGF2) forming floating cell aggregates named neurospheres [4,5,6]. NPCs from the SVZ of postnatal/adult animals have been used as cell therapy in different animal models of nervous system disorders, such as stroke, traumatic brain injury, spinal cord lesions, parkinsonism, or central axotomy, with generally reported beneficial results mainly attributed to bystander effects derived from the cell implant (i.e., immunomodulation, stimulation of angiogenesis, or neurotrophic support) [7,8,9,10,11,12]. The results obtained in animals support the possibility that the transplantation of NPCs could be a powerful strategy for regenerating human nervous system tissues damaged by disease or trauma. In this regard, the use of postnatal/adult NPCs might provide remarkable advantages compared to the use of embryonic NPCs, since it overcomes ethical issues derived from the use of embryos and it reduces the risk of tumor formation due to their lower proliferative capacity. However, this reduced proliferative activity also constitutes a limitation for their expansion in vitro. Another objection to the use of SVZ-derived adult NPCs in cell therapy is the reported low survival rate after implantation [10]. Therefore, research is being developed to enhance the lifespan and survival of this population of postnatal progenitor cells in order to achieve better functional outcomes after transplantation.

Purinergic receptors are widely expressed in the central nervous system, and they intervene in important biological processes both during development (with roles in NPC proliferation, differentiation, apoptosis, or neurotrophic factor synthesis and release) and in the adult brain (with roles in neurotransmission and neuromodulation) [13,14,15,16,17,18]. The purinergic receptor family includes two main receptor types: P1 receptors, sensitive to adenosine nucleosides, and P2 receptors, sensitive to nucleotides. There are four subtypes of P1 receptors: A_1_, A_2A_, A_2B_, and A_3_. P2 receptors are subdivided into ionotropic P2X receptors (P2X1-7), and metabotropic G protein-coupled P2Y receptors (P2Y1, 2, 4, 6, 11, 12, 13, and 14) [19]. In turn, nucleosides and nucleotides’ availability in the extracellular milieu can be regulated by extracellular ectonucleotidases [20]. Remarkably, neurogenic regions of the adult brain show higher ectonucleotidase activity than non-neurogenic regions [21,22,23], which suggests that purinergic signaling is important for the control of adult neurogenesis. Specifically in the SVZ, NPCs express all type of purinergic receptors (P1, P2X, and P2Y) [24,25,26,27,28] with the peculiarity that P2Y1 receptor expression is restricted to transit-amplifying intermediate progenitors (type C cells) and is lacking in SVZ neural stem cells (type B cells) [27]. In addition, SVZ NPCs are able to produce and release adenosine-5′-triphosphate (ATP) [21,29]. The effect of purinergic activation on cultured NPCs derived from the SVZ of adult mice has been tested, with differential reported results depending on the activated receptor subtype. For instance, the selective activation of P2Y receptors produces an inhibition in proliferative signals induced by EGF and FGF2 [24], whereas agonists for P2X receptors induce apoptosis and decrease NPC viability [26], which is an effect that has also been reported in other types of progenitor cells [30]. Based on this background, we aimed to explore the effects of a general blockade of purinergic signaling with suramin on the proliferation and survival of NPCs obtained from the SVZ of postnatal animals.

Our results show that suramin treatment significantly increases NPC survival by reducing apoptotic death without affecting the stemness properties of these cells such as their proliferation rate or their ability to generate neurons, astrocytes, and oligodendrocytes. Thus, we propose that the treatment of cultured NPCs with suramin is a valuable tool to enhance the survival of this population of progenitor cells before their therapeutic use.

## 2. Results

### 2.1. Suramin Treatment of NPC Cultures Induces an Increase in Neurosphere Size and in Cell Number Which is Not Attributable to a Higher NPC Proliferation Rate or to a Higher Incidence of Neurosphere Fusion

**Figure 1:**
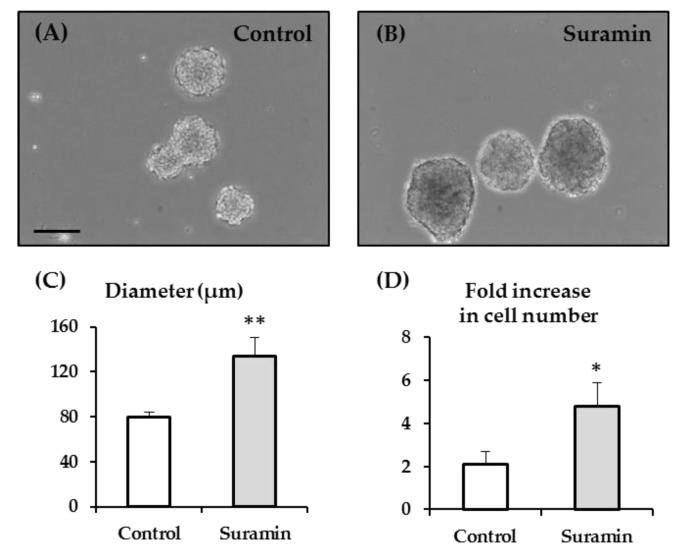
Effect of suramin on neurosphere size and on the number of viable neural progenitor cells. Neural progenitor cells (NPCs) of the postnatal rat subventricular zone were cultured in the absence (control) or presence of the purinergic receptor blocker suramin. The diameter of the formed neurospheres, as well as the number of viable cells after neurosphere disaggregation, was measured 72 h after seeding. (**A**,**B**) Phase-contrast photomicrographs of neurospheres in control cultures (**A**) and in cultures treated with 200 μM suramin (**B**). Bar: 100 μm. (**C**) Graph showing the neurosphere diameter (in μm) in each experimental condition. Data are the mean ± SEM (*n* = 8, ** *p* < 0.01; Student’s *t*-test). (**D**) Graph showing the fold increase relative to the initial number of seeded NPCs in each experimental condition. Data are the mean ± SEM (*n* = 8, * *p* < 0.05; Mann–Whitney Rank Sum Test).

We started by analyzing the effect of purinergic receptor blockade on the size of the neurospheres formed after the culture of NPCs isolated from the SVZ of postnatal rats maintained in proliferation conditions (in the presence of EGF and FGF2). For that purpose, we used suramin at 200 µM, which is a concentration in the range of previously reported actions as a non-selective purinergic receptor antagonist [21,31,32,33]. The treatment of NPC cultures with suramin for 72 h induced a marked increase in the diameter of the neurospheres (Figure 1A–C). Thus, neurospheres formed in NPCs treated with suramin presented a 1.67 times longer diameter than those formed in control conditions (133.97 ± 16.93 µm vs. 79.89 ± 4.66 µm; *p* < 0.01; Student’s *t*-test). Accordingly, the fold increase relative to the initial number of seeded cells was significantly higher in cultures treated with suramin compared to control cultures (4.8 ± 1.1-fold increase in suramin-treated cultures compared to 2.1 ± 0.6-fold increase in control cultures; *p* < 0.05; Mann–Whitney Rank Sum Test) (Figure 1D).

**Figure 2:**
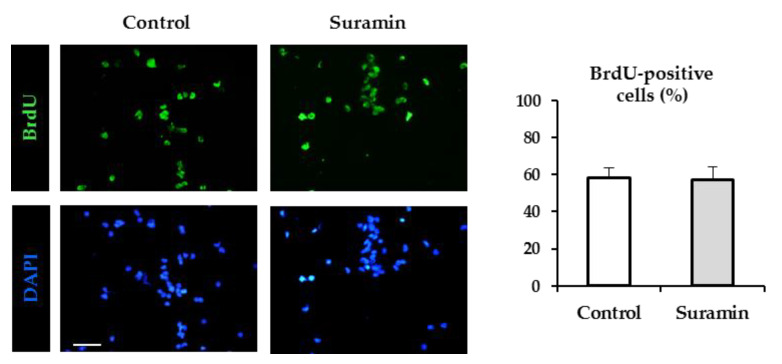
Effect of suramin on neural progenitor cell proliferation. Neural progenitor cells of the postnatal rat subventricular zone were cultured for 72 h in the absence (control) or presence of the purinergic receptor blocker suramin, and the percentage of bromodeoxyuridine (BrdU) incorporation during the last 12 h of culture was evaluated. Epifluorescence images showing BrdU immunohistochemistry (in green) in neurosphere-derived cells obtained from control cultures and cultures treated with 200 μM suramin. The total number of cells in each field was identified by 4′-6′-diamidino-2-phenylindole (DAPI) staining (in blue). Bar: 50 μm. The graph shows the percentage of BrdU-positive cells in each experimental condition. Data are the mean ± SEM (*n* = 6, *p* > 0.05, Mann–Whitney Rank Sum Test).

In order to analyze whether the increase in neurosphere diameter and cell number was due to an increase in cell proliferation, the thymidine analogue bromodeoxyuridine (BrdU) was added for the last 12 h of culture, and the number of proliferating cells was made visible by BrdU immunohistochemistry. Surprisingly, NPC culture treatment with 200 µM suramin did not significantly increase the number of BrdU-incorporating cells (Figure 2), which indicates that purinergic receptor blockade with suramin does not modify the proliferation rate of NPCs in SVZ neurospheres.

**Figure 3:**
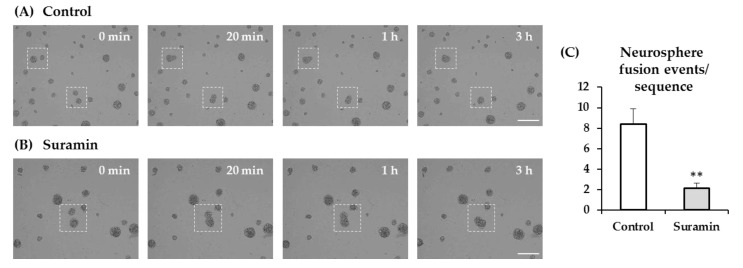
Effect of suramin treatment on neurosphere fusion. Neural progenitor cells of the postnatal rat subventricular zone were cultured for 72 h in the absence (control) or presence of 200 μM suramin. Time-lapse imaging was performed after 48 h of seeding over 21 h of culture in 10-min intervals. (**A**) Images captured from a time-lapse sequence showing neurospheres in control conditions. Dashed squares illustrate two examples of adjacent neurospheres that undergo adhesion (see *t* = 20 min) and fusion (*t* = 3 and 4 h). Bar: 200 μm. (**B**) Images captured from a time-lapse sequence showing neurospheres in a culture treated with 200 μM suramin. Dashed squares illustrate an example of adjacent neurospheres that undergo adhesion (see *t* = 20 min) and fusion (*t* = 3 and 4h). Bar: 200 μm. (**C**) Quantification of neurosphere fusion events per time-lapse sequence in each experimental condition (control or suramin). Data are mean ± SEM (*n* = 8, ** *p* < 0.01; Student’s *t*-test).

A higher incidence of aggregation between adjacent neurospheres might also account for the increased neurosphere size observed in suramin-treated cultures. To analyze this possibility, we performed time-lapse experiments in cultures treated or not with 200 µM suramin, and the number of neurosphere fusion events was counted in the recorded sequences. Neurosphere aggregation occurred at a lower rate in cultures treated with suramin than in control cultures, which excludes neurosphere fusion as a possible explanation for the effect of the treatment on neurosphere diameter (Figure 3).

### 2.2. Suramin Treatment Reduces Apoptosis in NPCs

**Figure 4:**
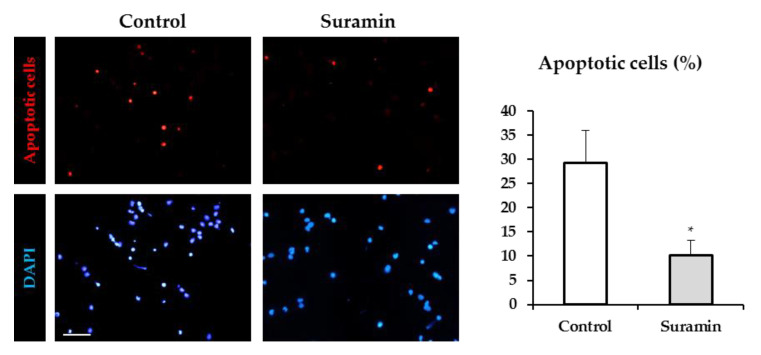
Effect of suramin treatment on neural progenitor cell apoptosis. Neural progenitor cells from the postnatal rat subventricular zone were cultured in the absence (control) or presence of 200 μM suramin, and the percentage of apoptotic cells was evaluated by the TUNEL assay after 72 h of culture. Epifluorescence images showing apoptotic cells (in red) in control cultures and in cultures treated with suramin. The total number of cells in each field was identified by DAPI staining (in blue). Bar: 50 μm. The graph shows the percentage of apoptotic cells in each experimental condition. Data are mean ± SEM (*n* = 8, * *p* < 0.05, Student’s *t*-test).

Cell survival is also an important factor that determines neurosphere size. To analyze whether suramin treatment may affect NPC survival, we evaluated the degree of apoptotic death by the terminal dUTP nick end labeling (TUNEL) assay in NPC cultures in the absence and presence of 200 μM suramin. The analysis of the number of apoptotic cells in both experimental conditions revealed that the percentage of apoptotic cells was strongly reduced by suramin treatment (Figure 4).

Therefore, we can conclude that purinergic receptor blockade with suramin induces a significant decrease in apoptosis, which might be responsible for the observed increase in cell number and neurosphere diameter.

### 2.3. Neural Lineage Potential is Not Affected in Neurospheres after Suramin Treatment

**Figure 5:**
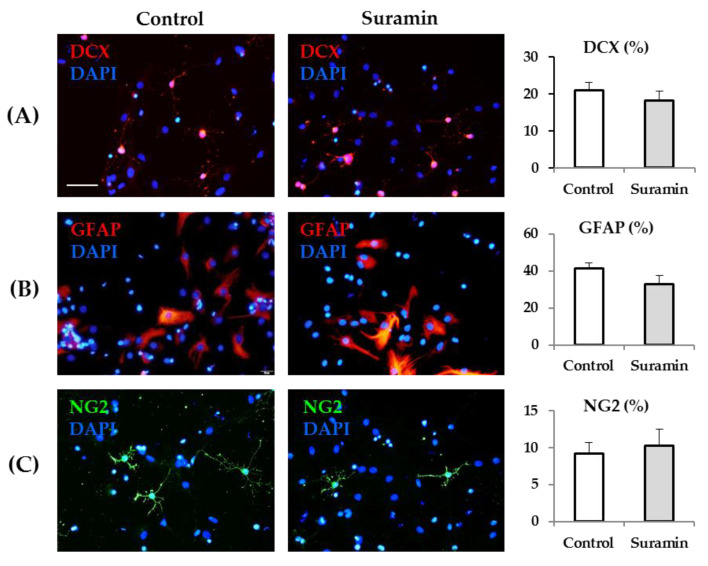
Neurosphere treatment with suramin does not modify neural progenitor cell fate. After 72 h of neural progenitor cell culture in the absence (control) or presence of suramin (200 μM), neurospheres were collected, dissociated, and induced to differentiate by seeding on an adhesive substrate in the absence of growth factors. Differentiation toward neurons, astrocytes, and oligodendrocyte precursors was evaluated seven days later. Representative epifluorescence images showing immunohistochemistry for the neuronal marker doublecortin (DCX, (**A**), in red), the astrocytic marker glial fibrillary acidic protein (GFAP, (**B**), in red), and the oligodendrocyte precursor marker neuron-glia antigen 2 (NG2, (**C**), in green) of cells from control (left column) or suramin-treated (middle column) cultures. The total number of cells in each field was identified by DAPI staining (in blue). Bar: 50 μm. The bar graphs show the quantification of the percentage of DCX-, GFAP-, or NG2-positive cells in each experimental condition. Data are the mean ± SEM (*n* = 6 for DCX, *n* = 4 for GFAP, *n* = 5 for NG2, *p* > 0.05, Student’s *t*-test).

Next, we aimed to investigate whether NPC treatment with suramin in proliferation conditions could modify cell multipotency. For that purpose, 72 h after control or suramin treatments, neurospheres were dissociated, and cells were seeded on an adhesive substrate to induce differentiation. Cells from control and suramin-treated neurospheres were maintained for 7 days in medium lacking growth factors and in the absence of any treatment, after which their phenotype was analyzed. As shown in Figure 5, the percentage of neurons (revealed by immunohistochemistry for doublecortin, DCX), astrocytes (revealed by immunohistochemistry for glial fibrillary acidic protein, GFAP), and oligodendrocyte precursors (revealed by immunohistochemistry for neuron-glia antigen 2, NG2) was very similar in NPCs derived from control and suramin-treated neurospheres.

### 2.4. The Levels of Connexin43 and Vascular Endothelial Growth Factor Increase in Neurospheres after Suramin Treatment

**Figure 6:**
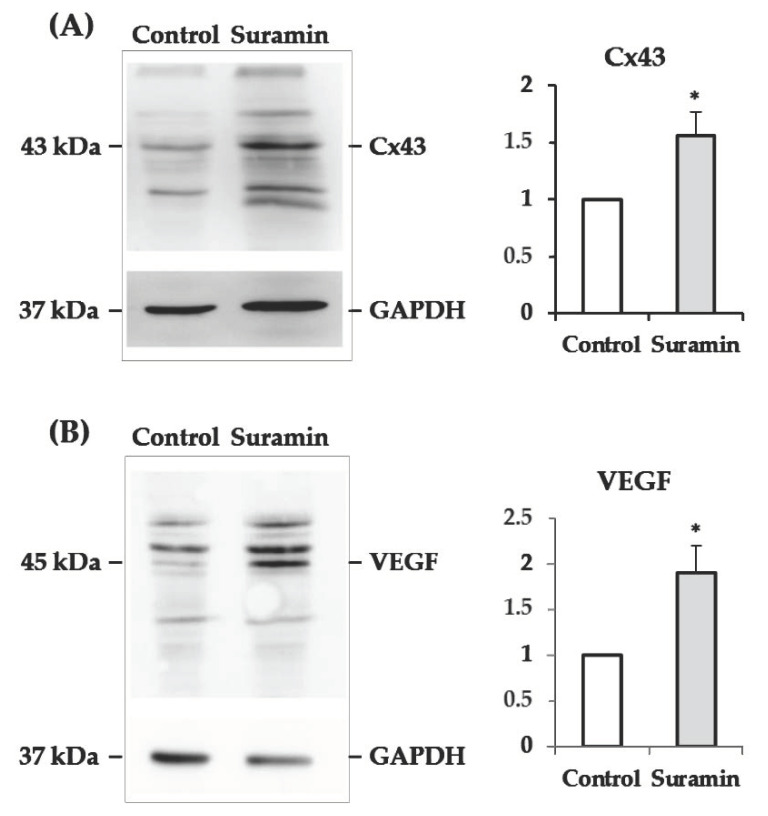
Effect of suramin treatment on connexin43 (Cx43) and vascular endothelial growth factor (VEGF) levels. Neural progenitor cells of the postnatal rat subventricular zone were cultured for 72 h in the absence (control) or presence of suramin (200 μM), and the levels of the hemichannel protein Cx43 and the growth factor VEGF were evaluated by Western blot. (**A**) Representative Western blot of Cx43 in protein extracts from control and suramin-treated neurospheres. The band corresponding to the monomeric form of Cx43 (43 kDa) is shown. Glyceraldehyde-3-phosphate dehydrogenase (GAPDH) immunoblotting was used as load control. Densitometry data (mean ± SEM) showed a significantly higher amount of Cx43 protein in treated neurospheres compared to control ones (normalized value of 1 for control; 1.56 ± 0.21 in suramin-treated neurospheres; *n* = 8, * *p* < 0.05, paired Student’s *t*-test). (**B**) Representative Western blot of neurosphere protein extracts showed a band of 45 kDa corresponding to the VEGF protein. GAPDH was used as load control. Densitometry data (mean ± SEM) showed a significantly higher amount of VEGF protein in suramin-treated neurospheres compared to control ones (normalized value of 1 for control; 1.90 ± 0.30 in suramin-treated neurospheres; *n* =3, * *p* < 0.05, paired Student’s *t*-test).

We aimed to seek possible explanations for the increased NPC survival derived from their treatment with suramin. Based on previous reports showing that coupling among adult SVZ-derived neurosphere cells by gap junctions is determinant for cell survival [34], we assessed whether the expression of the gap junction protein connexin43 (Cx43) was affected by the purinergic receptor blockade with suramin. Figure 6A shows that treatment with suramin induces a significant increase in the content of Cx43 in SVZ-derived neurospheres.

In addition, since NPCs are able to synthesize vascular endothelial growth factor (VEGF) [35], a molecule with neuroprotective and anti-apoptotic actions [36,37], we proceeded to analyze possible modifications in its expression derived from purinergic receptor blockade with suramin. As shown in Figure 6B, neurosphere treatment with suramin produced a 1.9-fold increase in VEGF protein, as revealed by Western blotting.

## 3. Discussion

In this study, we have demonstrated that the general blockade of purinergic receptors with suramin produces a significant increase in the survival of NPCs isolated from the SVZ of postnatal rats by reducing apoptotic cell death. Suramin treatment did not produce significant effects on NPC proliferation nor in their ability to generate neurons, astrocytes, or oligodendrocyte precursors. We have also shown that Cx43 and VEGF proteins are increased by neurosphere treatment with suramin and therefore, we suggest that these proteins might be involved in the anti-apoptotic actions derived from the purinergic receptor blockade.

NPCs of the postnatal SVZ have functional hemichannels by which nucleotides can be released to the extracellular space [38]. Indeed, SVZ NPCs have been shown to release ATP, both in vivo and in vitro [21,29]. Furthermore, the high ectonucleotidase activity identified in the SVZ is indicative of the importance of this signaling system for the adjustment of the extracellular levels of ATP to the precise needs of the niche [21,22,23]. In our study, we aimed to analyze the effect of purinergic receptor blockade on NPCs of the SVZ of postnatal animals cultured as neurospheres, which is a culture system devoid of any other cell components of the niche, that allows the analysis of possible autocrine effects of endogenously released nucleotides. The treatment of SVZ NPC cultures with suramin produces larger neurospheres and a higher number of viable cells. We excluded the possibility that this effect was due to an increased mitotic activity, since suramin failed to induce a higher degree of BrdU incorporation in NPCs. The longer neurosphere diameter was not due to neurosphere fusion, either. Quite the contrary, neurospheres formed in cultures treated with suramin showed a significantly lower tendency to fuse than those grown in its absence. Alternatively, the increased size of neurospheres might be attributed to survival-promoting effects derived from suramin treatment. To demonstrate this option, we performed TUNEL experiments that revealed that apoptosis resulted significantly reduced in NPCs treated with suramin. Therefore, we can conclude that by decreasing apoptosis, suramin induces an increase in NPC survival, which accounts for the increase in cell number and in neurosphere size observed in cultures treated with this purinergic antagonist. As neurogenesis requires a balance between the proliferation and subsequent death of surplus cells, our results suggest that ATP released by NPCs in neurospheres, by inducing apoptosis, might intervene in the maintenance of such balance. In agreement with this, other reports have shown that the activation of purinergic receptors (specifically P2X7 receptors) induces necrotic and apoptotic death of SVZ NPCs [26,39]. In these studies, purinergic agonists were used at concentrations that resemble the high ATP release produced during pathological conditions. Noteworthily, NPC proliferation is stimulated after several types of brain damage such as traumatic brain injury, hypoxia/ischemia, or epilepsy [40]. Thus, these authors have concluded that the P2X7 receptor intervenes in the regulation of excessive neurogenesis and/or gliogenesis produced after pathological conditions. In our experiments, the decrease in apoptosis found upon blockade of the signaling of ATP, endogenously released by NPCs in neurospheres, suggests that also in physiological conditions, ATP might intervene in the maintenance of adequate levels of neurogenesis to prevent uncontrolled proliferation.

The effects of purinergic activation on NPC proliferation have been associated to P2Y receptors, and results are variable depending on the experimental approach (in vivo or in vitro), the developmental stage, and the concentration of mitogens. For instance, two independent groups have shown that P2Y receptor activation in the SVZ in vivo stimulates NPC proliferation in the adult SVZ [27,41], which is an effect that has also been described in vitro in embryonic NPCs [21] and in adult NPCs cultured with low concentrations of EGF and FGF2 [42]. However, when the proliferation of adult NPCs is stimulated by EGF and FGF2, purinergic activation produces an inhibition of their mitotic activity [24]. The presence of additional cell types responding to purinergic signaling (as it happens in vivo), the activation or not of EGF and FGF2 receptor-mediated signaling, and the differential expression of P2YR in NPCs (type C cells express P2Y1R whereas type B cells do not) might explain these opposite results. In our experiments with postnatal SVZ neurospheres grown with EGF and FGF2, the blockade of the purinergic signaling did not modify NPC proliferation, as revealed by the analysis of BrdU incorporation. This result indicates that endogenously released ATP by postnatal SVZ-derived NPCs of neurospheres might not be relevant for the control of the cell cycle in this culture conditions. In addition, when suramin-treated NPCs were induced to differentiate, they gave rise to neurons, astrocytes, and oligodendrocyte precursors in the same proportion as untreated NPCs. The fact that suramin treatment does not modify either the proliferation rate or the NPC fate reinforces its putative usefulness as a survival-promoting agent for NPCs before their use in cell therapy.

Our experiments revealed that suramin treatment produced increased levels of the gap junction protein Cx43 in postnatal SVZ neurospheres. Cell coupling among neurosphere cells has been shown to be important for NPC survival. For instance, Ravella et al. reported that gap junction inhibition or Cx43 knockdown induced an increase in apoptosis in neurospheres obtained from the SVZ of postnatal mice [34]. Other authors have also reported beneficial effects on cell survival derived from neurosphere cell coupling [43,44]. We have previously demonstrated functional coupling by gap junctions in postnatal rat SVZ-derived neurospheres [38]. Based on this background, we suggest that the increase in Cx43 found in neurospheres treated with suramin might induce a higher degree of gap junctional coupling, which may intervene in the increased survival. Remarkably, when NPCs are implanted in lesioned tissue, they can form gap junctions with the host cells that are important for their survival and integration [45,46]. This leads us to hypothesize that treatment of NPCs with suramin after their implantation in the lesioned tissue might also increase their survival and integration in the host. Nevertheless, additional experiments will have to be carried out to corroborate this hypothesis.

We have previously reported that NPCs of the SVZ from postnatal cats and rats produce VEGF in vitro and after implantation in the lesioned tissue [12,35]. VEGF is an angiogenic factor with multiple neuroprotective functions in the nervous system, including anti-apoptotic actions (reviewed in [47]). Here, we show that suramin treatment of NPCs produces a noticeable increase in neurosphere VEGF content. The increased levels of this neuroprotective factor may also be responsible for the promotion in survival associated to the suramin treatment.

Therefore, we have demonstrated that NPC survival can be enhanced in vitro with suramin without inducing changes in proliferation or in phenotypic differentiation. Purinergic receptor blockade in SVZ neurospheres also leads to an increase in Cx43 and in VEGF. Thus, anti-apoptotic actions derived of gap junctional communication among neurosphere cells and/or of VEGF-derived neuroprotective actions might be involved in the survival-promoting effects of suramin. Our results are of interest for using suramin to improve NPC survival before their therapeutic use. The drug treatment allows the expansion of the NPC population without potentiating their mitotic activity, thus reducing its risk of tumorigenicity, and without modifying its differentiation pattern. In addition, we raise the possibility of using this drug after NPCs are implanted in the lesioned tissue to achieve better results on NPC survival and integration in the host tissue.

## 4. Materials and Methods

Experiments were performed with 7-day postnatal (P7) Wistar rats from both sexes (*Rattus norvegicus*) in accordance with the guidelines of the European Union (2010/63/EU) and Spanish law (R.D. 53/2013 BOE 34/11370-420, 2013) for the use and care of laboratory animals. Surgical procedures used in this study were approved by the ethics committee of Universidad de Sevilla.

### 4.1. Neurosphere Culture

NPCs were isolated from the SVZ of P7 Wistar rats of either sex and expanded in the form of neurospheres as follows. Four P7 rats were used for every independent culture. Briefly, the lateral walls of the lateral ventricles were removed and enzymatically dissociated with 1 mg/mL trypsin (Thermo Fisher Scientific, Waltham, MA, USA) at 37 °C for 15 min. Then, the tissue was centrifuged at 150 g for 5 min, rinsed in Dulbecco’s modified Eagle’s medium/F12 medium 1:1 (DF-12; Thermo Fisher Scientific) and centrifuged again in the same conditions. Then, the cells were resuspended in DF-12 containing 0.7 mg/mL ovomucoid (Sigma Aldrich, St. Louis, MO, USA) and mechanically disaggregated with a fire-polished Pasteur pipette. The dissociated cells were centrifuged and resuspended in defined medium (DM: DF-12 containing B-27 supplement minus vitamin A, 2 mM Glutamax^®^, 100 units/mL penicillin, 100 µg/mL streptomycin, and 0.25 µg/mL amphotericin B, all from Thermo Fisher Scientific), 20 ng/mL EGF (PeproTech, Rocky Hill, NJ, USA), and 10 ng/mL FGF2 (Millipore, Temecula, CA, USA). The cell suspension was maintained in T25 flasks, in an atmosphere of 5% CO_2_, at 37 °C. After 1–2 days, cell aggregates named neurospheres were formed. Neurosphere cells were subcultured every 3–4 days by centrifugation, mechanical dissociation, and resuspension with fresh DM. All the experiments were performed with neurosphere-derived cells between passages 2 and 6.

### 4.2. Analysis of Neurosphere Diameter, Bromodeoxyuridine (BrdU) Incorporation and Apoptosis

Every experiment was always performed in parallel with two T25 flasks seeded with neurosphere-derived cells from the same culture, at a density of 14,000 viable cells/cm^2^. One of the flasks received treatment with the purinergic receptor blocker suramin (200 μM, Sigma Aldrich, S2671), and the other flask (control) was treated with the vehicle (MilliQ^®^ water, Thermo Scientific, Waltham, MA, USA). Seventy-two hours after seeding, photographs of the neurospheres formed in the two flasks of every experiment (suramin and control) were captured using a Leica EC3 camera (Leica, Wetzler, Germany) coupled to a phase-contrast microscope (Leica DMIL-LED, Leica, Wetzler, Germany) with a 10× objective (eight photographs of random fields per flask). The diameter of neurospheres was measured with ImageJ (NIH). In every experiment, a mean value of neurosphere diameter was obtained for each condition. After photographs were taken, neurospheres in each flask (suramin and control) were collected, mechanically disaggregated, and counted by trypan blue exclusion to obtain the number of viable cells. This number was compared to the initial number of seeded cells in each flask in order to calculate the fold increase in cell number in each experimental condition.

For analyzing BrdU incorporation, experiments were performed as described above with the novelty that BrdU (Sigma Aldrich; 1 µM) was added to the flasks for the last 12 h of culture. Then, collected neurospheres from each flask (suramin and control) were slightly disaggregated with a P200 pipette and seeded on poly-L-ornithine-treated (Sigma-Aldrich) 12-mm diameter coverslips with DF-12 containing 1% fetal calf serum (FCS; Thermo Fisher Scientific) to allow adhesion. After 1 h, adhered cells were fixed with 4% paraformaldehyde in 0.1 M phosphate buffer (10-min incubation) and processed for BrdU immunohistochemistry. For that purpose, after several washes with phosphate-buffered saline (PBS), cells in the coverslips were exposed to a DNA denaturation treatment consisting of a 20-min incubation with 2N HCl. Subsequently, coverslips were immersed in borate buffer pH 8.5 for 10 min and then washed three additional times with PBS. Then, coverslips were incubated with a solution containing a BrdU antibody (Roche Diagnostics GmbH, Mannheim, Germany; Cat 11 170 376 001; 1:100) for 2.5 h, which was prepared in 2.5% bovine serum albumin (BSA; Sigma-Aldrich) in PBS. After rinsing, coverslips were incubated for 30 min with anti-mouse IgG coupled to FITC (Jackson ImmunoResearch, West Grove, PA, USA, 1:200), washed several times with PBS, and counterstained with 4′-6′-diamidino-2-phenylindole (DAPI, Sigma-Aldrich, 0.1 µg/mL) for 10 min. After final washes, coverslips were mounted on slides with an n-propyl-gallate solution (Sigma Aldrich, 0.1 M) prepared in glycerol:PBS 9:1.

To analyze cell death by apoptosis, collected neurospheres after 72 h of culture were disaggregated and seeded on poly-L-ornithine-treated 12-mm diameter coverslips with DF-12 containing 1% FCS. After 1 h of seeding, coverslips were fixed for 15 min with 4% paraformaldehyde in 0.1 M phosphate buffer and processed for terminal dUTP nick end labeling (TUNEL) assay using the instructions provided in the kit (Molecular Probes, Click-iT^®^ Plus TUNEL Assay C10618).

### 4.3. Time-Lapse Microscopy

For analyzing neurosphere fusion during the time of culture, some experiments were performed in dishes designed for the high-end microscopy visualization of living cells (35 mm, high 60 μ-Dish, Ibidi, Germany) seeded with neurosphere-derived cells at a density of 14,000 viable cells/cm^2^. Dishes were treated with 200 μM suramin or with MilliQ^®^ water at the time of seeding and maintained in an atmosphere of 5% CO_2_ at 37 °C in an incubator. Forty-eight hours after seeding, dishes were transferred to a Zeiss Apotome microscope (Zeiss, Oberkochen, Germany) equipped with a temperature-controlled 37 °C chamber and a gas controlled system providing a continuous 5% CO_2_ stable atmosphere and 100% humidity in the chamber. The whole microscope was enclosed in a big incubator (Pecon Heating Unit XL), minimizing thermal drift for long experiments. For axial drift correction, an autofocus routine with a parallel infrared 835 nm LED (Definite Focus) was run automatically at the beginning of each time point. Four positions in each dish were selected, and photographs (10×) in each position were taken every 10 min for a total period of 21 h. Transmitted light images were taken with a monochrome CCD camera (Axiocam 506) coupled to the microscope. Neurosphere fusion events were analyzed in time-lapse image sequences using the ZEN 2011 software (Zeiss, Oberkochen, Germany).

### 4.4. Analysis of Differentiation of Neurosphere-Derived Cells

To analyze differentiation, neurospheres obtained from T25 flasks treated for 72 h with 200 μM suramin or with MilliQ^®^ water (Thermo Scientific) (control) were mechanically dissociated and seeded on poly-L-ornithine-treated (Sigma Aldrich) 12-mm diameter coverslips in DF-12 with 1% FCS at a density of 10,000 cells/coverslip. After 4 h, adhered cells in the coverslips were washed, and the following medium devoid of growth factors was added: DF-12 containing B-27 supplement, 2 mM Glutamax^®^, 100 units/mL penicillin, 100 µg/mL streptomycin, and 0.25 µg/mL amphotericin B (Thermo Fisher Scientific). After seven days, coverslips were fixed for 10 min with 4% paraformaldehyde in 0.1 M phosphate buffer. Coverslips were incubated for 30 min in a blocking solution containing 2.5% BSA in PBS and then in the primary (2 h at room temperature)—and, after rinsing, in the secondary (30 min at room temperature)—antibodies prepared in blocking solution and in PBS, respectively. After washing, cells were counterstained with 0.1 µg/mL DAPI for 10 min, washed again, and mounted on slides with the n-propyl-gallate solution described earlier. The primary antibodies used were doublecortin (DCX, to identify neurons, Santa Cruz Biotechnology, Santa Cruz, CA, USA, sc-271390, 1:100), glial fibrillary acidic protein (GFAP, to identify astrocytes, Sigma Aldrich, G3893, 1:1,400), and neuron-glia antigen 2 (NG2, to identify oligodendrocyte precursors, Merck Millipore, Darmstadt, Germany, AB5320, 1:400). The secondary antibodies used were: anti-mouse IgG labeled with TRITC and anti-rabbit IgG labeled with FITC (Jackson ImmunoResearch, West Grove, PA, USA, 715-025-150 and 711-095-152, respectively, 1:200).

### 4.5. Epifluorescence Microscopy

Fluorescent images of the cells in coverslips were captured using a camera DP73 (Olympus, Hamburg, Germany) coupled to an epifluorescence microscope (Olympus BX61) with a 20× objective. The omission of primary antibodies resulted in the absence of detectable staining in all cases. Six random fields were captured per coverslip with the excitation wavelengths for FITC, TRITC, or Alexa Fluor^TM^ 594 (the fluorophore of the TUNEL kit), and for DAPI.

Counting of BrdU-positive cells and TUNEL-positive cells was performed in the images and expressed as a percentage of the total number of cells identified by DAPI staining.

Counting of DCX-, GFAP-, or NG2-positive cells was performed on the merged images of the marker with the DAPI channel. Only cells with clear labeling of the cytoplasm or the processes around the DAPI labeled nuclei were considered immunopositive for each marker.

### 4.6. Western Blot

Western blot experiments were performed to evaluate Cx43 and VEGF expression in neurospheres. Every experiment was performed in parallel with two T25 flasks seeded with neurosphere-derived cells from the same culture at a density of 14,000 viable cells/cm^2^. One of the flasks received 200 μM suramin treatment, and the other flask (control) was treated with MilliQ^®^ water. After 72 h of culture, neurospheres from each flask were collected, washed twice in PBS, and centrifuged for 5 min at 900 rpm. The pellet was homogenized in ice-cold lysis buffer containing a cocktail of proteases inhibitors (Merck, Darmstadt, Germany; 11836170001) for 20 min with occasional gentle shaking. Then, the suspension was centrifuged for 30 min at 13,000 rpm and 4 °C. Total protein concentration was determined in the supernatant by the Bradford method [48], using BSA as a standard. Proteins were diluted in sample buffer (62.5 mM Tris HCl, pH 6.8, 10% glycerol, 10% SDS, 5% β-mercaptoethanol, 0.05% bromophenol blue), denatured at 95 °C for 6 min, and then separated by 15% SDS PAGE (50 μg/lane) and transferred to polyvinylidene difluoride (PVDF) membranes by electroblotting. After the blockage of unspecific antigens with 5% BSA for 1 h, blots were incubated overnight at 4°C in a solution with antibodies raised in rabbit against either Cx43 (Thermo Fisher Scientific; 71-0700; 1:200) or VEGF (ABCAM, Cambridge, MA, USA; AB-46154; 1:1000) diluted in Tris-Buffered Saline (TBS) with 0.1% Tween supplied with 5% BSA. Then, membranes were incubated in a solution containing the HRP-conjugated anti-rabbit secondary antibody (Vector Labs, Burlingame, CA, USA; PI-1000; 1:10000) prepared in TBS-Tween 0.1% for 90 min at room temperature. The immunoreactions were detected using the WesternBright Quantum kit (Advansta, Menlo Park, CA, USA; K-12042). Images from the bands were visualized using a chemiluminescence imaging system (Fusion-Solo S, Vilber, France). After washing the membranes for 10 min with stripping buffer, blots were reprobed with anti-glyceraldehyde-3-phosphate dehydrogenase (GAPDH) mouse monoclonal antibody (Millipore; MAB374; 1:1000), followed by HRP-conjugated anti-mouse secondary antibody (Vector Labs, Burlingame, CA, USA; PI-2000; 1:10,000), to ensure equal loading. Bands were analyzed using Multi-Gauge v3.0 software (Fujifilm, Tokyo, Japan). For each lane, data were expressed relative to GAPDH values after background subtraction. Data were relativized to the values obtained in control neurospheres.

### 4.7. Statistics

Statistics were performed with Sigma Plot 11 (Systat Software, San José, CA, USA). All data are expressed as the mean ± standard error of the mean (SEM).

Comparisons between the control and the treatment groups were achieved using the Student’s *t*-test for parametric or Mann–Whitney Rank Sum test for non-parametric data except for data obtained from Western blot experiments that were compared using a paired *t*-test (overall level of significance *p* < 0.05).

## 5. Patents

Patent ES2734733 (“Procedure to extend the lifespan of neonatal neural progenitor cells with suramin”).
